# Factors associated with intervertebral cage subsidence in posterior lumbar fusion

**DOI:** 10.1186/s13018-023-04479-w

**Published:** 2024-01-03

**Authors:** Yan Liu, Nian-Hu Li

**Affiliations:** 1 Shandong University of Traditional Chinese Medicine, Jinan, China; 2 Department of Orthopedics, Affilited Hospital of Shandong University of Traditional Chinese Medicine, Jinan, China

**Keywords:** Posterior lumbar interbody fusion, Cage subsidence, Multi-factor correlation analysis

## Abstract

**Background:**

The interbody fusion apparatus is a key component of the operation and plays a key role in the postoperative efficacy. Cage subsidence is one of the common complications after lumbar fusion and internal fixation. Clinical studies on the risk factors of cage subsidence are incomplete and inaccurate, especially paravertebral muscle atrophy and intervertebral bone fusion time.

**Methods:**

Among the patients who underwent PLIF surgery in our hospital from January 2016 to January 2019, 30 patients with cage subsidence and 30 patients without cage subsidence were randomly selected to be included in this study. The differences between the two groups were compared, and the relevant factors of cage subsidence were explored by single factor comparison and multiple logistic regression analysis.

**Results:**

Bone mineral density (T) of the subsidence group [(− 1.84 ± 1.81) g/cm^2^ vs (− 0.87 ± 1.63) g/cm^2^, *P* = 0.018] was significantly lower than that of the normal group. There were 4 patients with end plate injury in the subsidence group (*P* = 0.038). Preoperative end plate Modic changes [I/II/III, (7/2/2) vs (2/5/8), *P* = 0.043] were significantly different between the two groups. In the subsidence group, preoperative rCSA of psoas major muscle [(1.43 ± 0.40) vs (1.64 ± 0.41), *P* = 0.043], CSA of paravertebral muscle [(4530.25 ± 776.55) mm2 vs (4964.75 ± 888.48) mm^2^, *P* = 0.047], paravertebral muscle rCSA [(3.03 ± 0.72) vs (3.84 ± 0.73), *P* < 0.001] and paravertebral muscle rFCSA [(2.29 ± 0.60) vs (2.89 ± 0.66), *P* < 0.001] were significantly lower than those in normal group. In the subsidence group, the vertebral body area [(1547.81 ± 309.89) mm2 vs (1326.48 ± 297.21) mm2, *P* = 0.004], the height of the immediately corrected vertebral space [(2.86 ± 1.10) mm vs (1.65 ± 1.02) mm, *P* = 0.020], immediately SL corrective Angle [(5.81 + 4.71)° vs (3.24 + 3.57) °, *P* = 0.009), postoperative PI—LL [(11.69 + 6.99)° vs (6.66 + 9.62) °, *P* = 0.029] and intervertebral fusion time [(5.38 ± 1.85) months vs (4.30 ± 1.49) months, *P* = 0.023] were significantly higher than those in the normal group. Multivariate logistic regression analysis showed that the time of intervertebral fusion (OR = 1.158, *P* = 0.045), the height of immediate intervertebral space correction (OR = 1.438, *P* = 0.038), and the Angle of immediate SL correction (OR = 1.101, *P* = 0.019) were the risk factors for cage subsidence. Bone mineral density (OR = 0.544, *P* = 0.016) and preoperative paravertebral muscle rFCSA (OR = 0.525, *P* = 0.048) were protective factors.

**Conclusion:**

Intervertebral fusion time, correctable height of intervertebral space, excessive Angle of immediate SL correction, bone mineral density and preoperative paravertebral muscle rFCSA are risk factors for cage subsidence after PLIF.

## Introduction

Lumbar interbody fusion is a recognized treatment for a variety of spinal disorders, including trauma, infection, and tumors. It involves placing an implant (spacer, bone graft, or fusion apparatus) in the intervertebral space after discectomy. Often, the decision to fuse lumbar segments is based on instability or severe symptomatic degeneration. The main goals of lumbar fusion include symptom relief and segmental stabilization. Posterior lumbar interbody fusion (PLIF) is the most common surgical method in lumbar interbody fusion and plays an important role in lumbar surgery. The posterior spinal approach avoids vascular and nerve damage associated with the anterior approach and allows a single incision for bilateral decompression and interbody fusion. Compared with other fusion techniques, PLIF has a wide surgical field of view and is less difficult to operate, and the fusion rate during follow-up is better. It also has good clinical results, including pain reduction, but the disadvantages of PLIF include large open incisions, large blood loss, and a long surgical time, which are likely to cause neuro-related complications. Complications associated with this approach include adjacent segment degeneration, failure of internal fixation, fusion cage displacement, and foraminal stenosis due to fusion cage subsidence. Current studies on PLIF focus on improving the effect of surgery while avoiding complications.

At present, surgical treatment is a better choice for patients with lumbar degenerative changes who have failed conservative treatment, and the surgical effect of posterior lumbar surgery is worthy of recognition (1). The interbody fusion apparatus is a key component of the operation and plays a key role in the postoperative efficacy. Fusion instrument subsidence is one of the common complications after lumbar fusion and internal fixation. In recent years, the incidence of fusion instrument subsidence after posterior lumbar fusion surgery has been reported to be 26–50%, mostly within 1 year after surgery (2). cage subsidence appears on radiographs as the fusion implant is embedded into the end plate or cancellar bone, resulting in loss of intervertebral height, reduced anterior column support, reduced local lordosis and lumbar lordosis, leading to progressive spinal deformities, neurological deterioration, and non-fusion (3). In severe cases, secondary surgery is required. It is very important to treat the high risk factors of fusion device subsidence in advance and reduce the probability of subsidence for the success of surgery. Clinical studies on the risk factors of cage subsidence are often incomplete and inaccurate, especially paravertebral muscle atrophy and interbody bone fusion time were not included in the analysis. Therefore, the purpose of our study was to comprehensively explore the risk factors of cage subsidence, conduct correlation analysis of risk factors, and clarify the independent risk factors of fusion and their critical thresholds.

## Information and methods

### General information

From January 2016 to January 2019, patients who underwent L4/5 single-stage posterior decompression interbody bone graft fusion and internal fixation were followed up in Shandong Hospital of Traditional Chinese Medicine (the surgery was performed by experienced surgeons with the same years of experience). 30 patients with cage subsidence and 30 patients without cage subsidence were randomly selected and divided into subsidence group and normal group. In the subsidence group, there were 30 cases, including 11 males and 19 females, aged 56.95 ± 10.23 years. The follow-up time was 30.34 ± 5.19 months. The bone mineral density was − 1.84 ± 1.81 g/cm2. 2; In the normal group, there were 30 cases, 14 males and 16 females, aged 52.91 ± 12.97 years. The follow-up time was 28.98 ± 4.79 months. The bone mineral density was − 0.87 ± 1.63 g/cm2. This study was approved by the hospital Ethics Committee and all patients gave their informed consent.

Inclusion criteria: (1) Patients underwent L4/5 single-level surgery for lumbar degenerative diseases (lumbar spinal stenosis, lumbar disc herniation, lumbar spondylolisthesis); (2) The surgical method was posterior lumbar interbody fusion; The clinical data and follow-up data were complete and the follow-up time was at least 2 years. (4) Voluntarily accept surgical treatment and sign informed consent.

Exclusion criteria: (1) multi-segmental surgery; (2) the patient did not have scoliosis, kyphosis and other factors; (3) postoperative infection, broken nails and rods; (4) the patient had a history of spinal surgery for various reasons; (5) patients with tuberculosis, tumor, compulsory spondylitis and other special diseases; (6) patients suffering from mental illness, depression, etc., who do not cooperate with treatment; (7) The follow-up data were incomplete.

### Surgical methods

After successful general anesthesia, the patient was placed in a prone position and the patient's abdomen was suspended. C-arm fluoroscopy was used to locate the pedicle body surface projection of the vertebral body during the operation, and a median incision was made at the back of the waist. The electroknife was removed layer by layer to the lamina, and the facet joints and transverse process were expanded to both sides. The insertion point of the pedicle screw was located, the positioning needle was placed, and the accurate position was confirmed by C-arm perspective. The orientation of the positioning needle was twisted into the pedicle nail, and the spinal canal decompression was performed. Directly, the nucleus pulposus forceps removed the prolapsed nucleus pulposus tissue. In addition, the lateral recess and nerve root canal were enlarged, and the nerve root lysis was complete. After the nucleus pulposus tissue was removed, the cartilaginous end plate was alternately scraped with a tooth scraper, and the interdisk tissue was completely removed. The soft tissue of the bitten lamina was removed and then trimmed into granular bone pieces and implanted into the vertebral space. The interbody fusion cage of appropriate size was inserted into the space, and the Cage was filled with bone fragments. Place the pre-bent titanium rod at the nail tail and tighten the nut to cut off the tail. Check instruments, built-in drainage tube, layer by layer suture.

### Observation indicators

General information: Age, sex, BMI, course of disease, follow-up time, disease diagnosis, diseased segment, history of alcohol abuse, smoking history, history of hypertension, history of diabetes and bone mineral density measured by ultrasound were recorded in both groups.

Imaging data: Cross sectional area (CSA), vertebral body CSA, paravertebral muscle CSA, functional paravertebral muscle cross sectional area (FCSA) and paravertebral fat CSA were measured before operation using Image J software (National Institutes of Health, USA). The gray threshold was set to 120, and the percentage of pixels representing fat in paravertebral CSA was calculated, namely fat infiltration degree (5). Relative lumbar major cross sectional area (rCSA), paravertebral rCSA, relative functional paravertebral cross sectional area (rFCSA), and fat infiltration degree (fat infiltration, FI) were calculated. rCSA, that is, the ratio of muscle CSA at the same level to disc CSA (changed to vertebral CSA due to disc herniation) was calculated to control the influence of body type, weight, and height on muscle CSA (6). The patient's endlaminitis was recorded before surgery, and the intraoperative SL correction Angle, intraoperative vertebral space height, PT, LL, SS and PI-LL were measured 2 days after surgery. CT scan was performed periodically after surgery to observe the intervertebral fusion. The success of bone fusion could be judged by the continuous bone bridge connecting vertebral cartilage end plate on CT film, and the intervertebral fusion time of each patient was recorded.

Perioperative data: Operation time, incision length, intraoperative blood loss, intraoperative end plate injury, time of departure, and complications were recorded. Lumbar pain Scale (VAS) and Oswestry Disability Index (ODI) were evaluated for all patients before surgery, 2 days after surgery, 3 months after surgery, and at the last follow-up. The time of postoperative intervertebral fusion, postoperative adjacent level degeneration, and failure of internal fixation were recorded.

### Statistical analysis

SPSS20.0 software was used for statistical analysis. Measurement data were represented by (x ± s). When data were normally distributed, independent sample t test was used for comparison between the two groups. When the data is not normally distributed, the rank sum test is used. Counting data were tested by 2 test or Fisher exact test. Mann–whitney U test was used to compare the grade data between the two groups. *P* < 0.05 was considered statistically significant. Multivariate logistic regression analysis was performed for statistically significant indicators to determine the independent risk factors for fusion subsidence after PLIF. Intra-class correlation (ICC) was used to assess the agreement between the two observers, ICC is equal to the individual variability divided by the total variability, so its value is between 0 and 1. 0 indicates untrusted and 1 indicates fully trusted. It is generally believed that a reliability coefficient lower than 0.4 indicates poor reliability, and a reliability coefficient greater than 0.75 indicates good reliability.

## Results

### Consistent results

In order to evaluate the inter-observer and inter-observer consistency, ICC values were calculated. The intra-observer ICC values and inter-observer ICC values were 0.869 and 0.834, respectively, showing a good consistency.

### General information

There were no statistically significant differences between the two groups in age, sex, BMI, course of disease, follow-up time, disease diagnosis, pathological segment, history of alcohol abuse, smoking history, history of hypertension and history of diabetes, etc. There were significant differences in bone mineral density and the number of osteoporosis patients between the two groups (*P* = 0.018, *P* = 0.028) (Table 1). The bone mineral density in the subsidence group was significantly lower than that in the normal group, and the number of osteoporosis patients in the subsidence group was significantly higher than that in the normal group.

**Table 1:** Univariate comparison between the subsidence group and the non-sunk group

Index	subsidence group (*n* = 30)	normal group (*n* = 30)	*P* values
*General information*			
Age (years, $\overline{x}$ ± s)	56.95 ± 10.23	52.91 ± 12.97	0.195
Gender (e.g., male/female)	11/19	14/16	0.432
BMI (kg/m^2^, $\overline{x}$ ± *s*)	25.41 ± 6.74	25.30 ± 2.92	0.913
Alcoholic (e.g., yes/no)	5/25	1/29	0.197
Smoking (e.g., yes/no)	7/23	2/28	0.148
Diabetes (e.g., yes/no)	4/26	7/23	0.505
Hypertension (e.g., yes/no)	13/17	9/21	0.422
Osteoporosis (e.g., yes/no)	10/20	3/27	0.028
Bone mineral density (T value, g/cm^2^, $\overline{x}$ ± s)	− 1.84 ± 1.81	− 0.87 ± 1.63	0.018
Duration of disease (months, $\overline{x}$ ± s)	5.67 ± 5.13	6.21 ± 3.98	0.376
Etiology (e.g., DH/SS/LS)	13/10/7	9/9/12	0.351
Follow-up time (months, $\overline{x}$ ± s)	30.34 ± 5.19	28.98 ± 4.79	0.793
*Image data*			
End plate Modic change (e.g., I/II/III)	7/2/2	2/5/8	0.043
Postoperative LL (º, $\overline{x}$ ± s)	40.01 ± 11.25	42.43 ± 9.37	0.984
Postoperative PT (º, $\overline{x}$ ± s)	20.28 ± 7.27	17.13 ± 9.89	0.181
Postoperative SS (º, $\overline{x}$ ± s)	30.54 ± 9.55	31.89 ± 7.23	0.491
Postoperative PI-LL (º, $\overline{x}$ ± s)	11.69 ± 6.99	6.66 ± 9.62	0.029
Preoperative vertebral space height (mm, $\overline{x}$ ± s)	9.20 ± 1.76	8.68 ± 1.62	0.212
Preoperative psoas major muscle CSA (mm^2^, $\overline{x}$ ± s)	2226.90 ± 700.27	2149.01 ± 646.44	0.637
Preoperative rCSA of psoas major ($\overline{x}$ ± s)	1.43 ± 0.40	1.64 ± 0.41	0.043
Preoperative paravertebral muscle CSA (mm^2^, $\overline{x}$ ± s)	4530.25 ± 776.55	4964.75 ± 888.48	0.047
Preoperative paravertebral muscle rCSA ($\overline{x}$ ± s)	3.03 ± 0.72	3.84 ± 0.73	< 0.001
Preoperative vertebral body CSA (mm^2^, $\overline{x}$ ± s)	1547.81 ± 309.89	1326.48 ± 297.21	0.004
Preoperative paravertebral muscle FI (%, $\overline{x}$ ± s)	24.29 ± 6.23	24.67 ± 8.40	0.770
Preoperative paravertebral fat CSA (mm^2^, $\overline{x}$ ± s)	559.48 ± 197.30	630.39 ± 219.32	0.258
Preoperative paravertebral muscle FCSA (mm^2^, $\overline{x}$ ± s)	3434.61 ± 689.50	3751.29 ± 884.98	0.137
Preoperative paravertebral muscle rFCSA ($\overline{x}$ ± s)	2.29 ± 0.60	2.89 ± 0.66	< 0.001
Intervertebral fusion time (months, $\overline{x}$ ± s)	5.38 ± 1.85	4.30 ± 1.49	0.023
Immediate correction of vertebral space height (mm, $\overline{x}$ ± s)	2.86 ± 1.10	1.65 ± 1.02	0.020
Immediate SL correction Angle (°, $\overline{x}$ ± s)	5.81 ± 4.71	3.24 ± 3.57	0.009
Fusion settling height (mm, $\overline{x}$ ± s)	2.87 ± 0.77	0.93 ± 0.53	< 0.001
Adjacent stage degeneration (e.g., up/down/down)	7/4/0	6/2/1	0.605
Failure of postoperative internal fixation	2	0	0.150
*Surgical data*			
Operation time (h, $\overline{x}$ ± s)	1.92 ± 0.33	1.94 ± 0.32	0.863
Total incision length (cm, $\overline{x}$ ± s)	10.57 ± 1.86	10.10 ± 1.92	0.309
Intraoperative blood loss (ml, $\overline{x}$ ± s)	288.10 ± 86.47	266.18 ± 90.79	0.331
Intraoperative end plate injury (e.g., yes/no)	4/26	0/30	0.038
Ground travel time (d, $\overline{x}$ ± s)	2.76 ± 0.70	2.74 ± 0.59	0.863
Wound healing (e.g., A/B/C)	29/1/0	30/0/0	0.313
Length of stay (d, $\overline{x}$ ± s)	10.62 ± 2.18	10.82 ± 2.47	0.738
Follow-up data			
*VAS score for low back pain*			
Before operation	7.43 ± 0.60	7.57 ± 0.63	0.354
2 days after surgery	5.29 ± 1.01	4.84 ± 0.64	0.066
3 months after surgery	2.05 ± 0.67	2.06 ± 0.64	0.945
Last follow-up	1.33 ± 0.80	1.35 ± 0.97	0.933
*ODI score*			
Before operation	47.11 ± 5.87	48.28 ± 8.06	0.538
2 days after surgery	34.71 ± 4.11	35.61 ± 4.98	0.456
3 months after surgery	24.29 ± 4.68	24.24 ± 4.15	0.962
Last follow-up	15.95 ± 5.15	16.25 ± 4.85	0.809

### Perioperative situation

All operations were successfully completed. One patient in the subsidence group had delayed incision healing, while the other patients had no abnormal incision, and all patients were healed. The perioperative data are shown in Table 1. After comparative analysis, there were 4 patients with end plate injury in the subsidence group (*P* = 0.038). There were no significant differences in operation time, incision length, intraoperative blood loss, ground time, hospital stay and walking time between the two groups (*P* > 0.05) (Table 1).

### Preoperative degeneration of paravertebral muscle and psoas major muscle

**Fig. 1:**
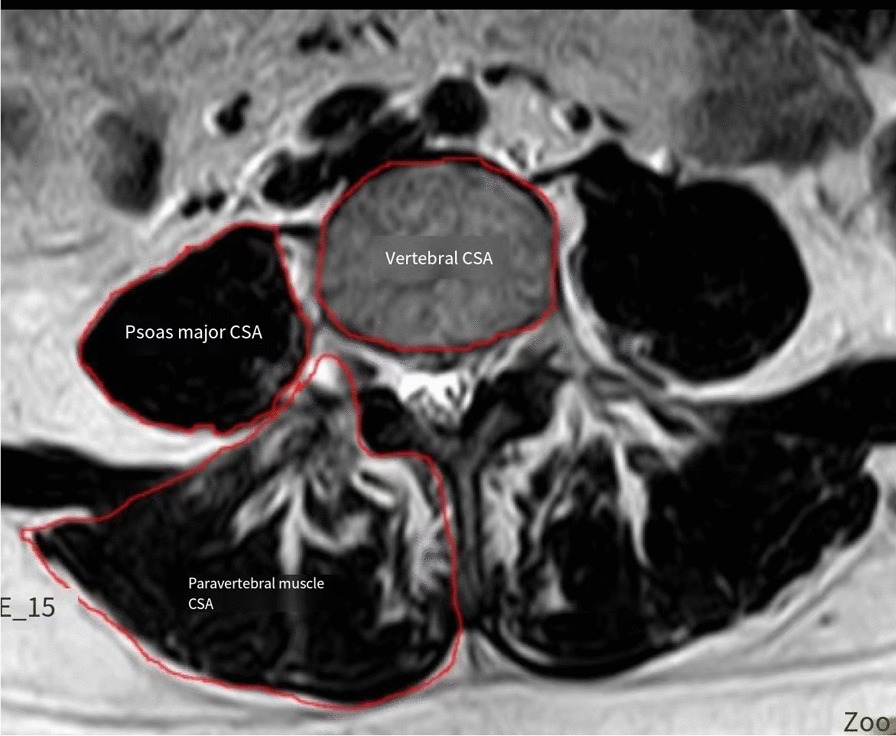
Schematic diagram of muscle measurement

rCSA of psoas major muscle, CSA of paravertebral muscle, rCSA of paravertebral muscle and rFCSA of paravertebral muscle in subsidence group were significantly lower than those in normal group, with statistical significance (*P* = 0.043, *P* = 0.047, *P* < 0.001, *P* < 0.001). CSA in the subsidence group was significantly higher than that in the normal group, and the difference was statistically significant (*P* = 0.004). There were no significant differences in psoas major muscle CSA, paravertebral muscle FI, paravertebral muscle fat CSA and paravertebral muscle FCSA between the two groups (*P* > 0.05). See Fig. 1 for specific data (Table 1).

### Visual data

The subsidence height of the fusion in the subsidence group was (2.87 ± 0.77) mm, and the subsidence height of the fusion in the normal group was (0.93 ± 0.53) mm. The intervertebral fusion time in the subsidence group was significantly longer than that in the normal group, with statistical significance (*P* = 0.023). The height of immediately corrected intervertebral space in the subsidence group was significantly higher than that in the normal group, and the difference was statistically significant (*P* = 0.020). The immediate SL correction Angle in the subsidence group was significantly higher than that normal group, and the difference was statistically significant (*P* = 0.009). The difference of PI-LL in the subsidence group was significantly higher than that in the normal  group (*P* = 0.029). In the subsidence group, there were 2 cases of type I, 10 cases of type II and 8 cases of type III. In the normal group, there were 3 cases of type I, 8 cases of type II and 1 case of type III, and the difference was statistically significant (*P* = 0.043). There were no significant differences in preoperative L4/5 intervertebral space height, postoperative LL, postoperative PT, postoperative SS, postoperative internal fixation failure and adjacent segment degeneration between the two groups (*P* > 0.05). See Fig. 1 for specific data (Table 1).

### Follow-up data

There were no significant differences in VAS scores and ODI scores between the two groups before surgery, 2 days after surgery, 3 months after surgery and at the last follow-up (*P* > 0.05), and the specific data are shown in Table 1.

### Binary multi-factor logistic regression analysis

Multivariate logistic regression analysis showed that the classification ability of the model was 87.7%, and the Chi-square test model was valid (× 2 = 5.857, *P* = 0.663). The time of intervertebral fusion (OR = 1.158, *P* = 0.045), the height of immediate intervertebral space correction (OR = 1.438, *P* = 0.038), and the Angle of immediate SL correction (OR = 1.101, *P* = 0.019) were the risk factors of cage subsidence. Bone mineral density (OR = 0.544, *P* = 0.016) and paravertebral muscle rFCSA (OR = 0.525, *P* = 0.048) were protective factors for the cage subsidence (Table 2).

**Table 2:** Results of multi-factor logistic regression analysis of cage subsidence

Influencing factor	B value	S.E	Wald value	OR value	95% CI	*P* value
Psoas major rCSA	− 0.069	0.583	2.513	0.933	0.804–7.889	0.475
Intervertebral fusion time	0.147	3.22	4.002	1.158	0.832–1.962	0.045
Paravertebral muscle rFCSA	− 0.644	0.931	3.107	0.525	0.280–0.987	0.048
Bone mineral density	− 0.609	0.151	5.760	0.544	0.305–0.968	0.016
Immediate vertebral space correction height	0.363	0.294	4.290	1.438	1.069–1.935	0.038
Instant SL correction height	0.096	0.094	5.521	1.101	1.019–1.323	0.019
Postoperative PI-LL	− 0.008	0.039	3.208	1.008	0.863–1.007	0.073

## Discussion

Posterior lumbar fusion surgery (PLIF) is a very mature technique that has been applied to clinical treatment by many spine surgeons (7). It is suitable for patients with minimally invasive and difficult operation and complicated conditions, and full visual field exposure can ensure the safety and operability of surgery. Today, PLIF and related fusion methods are the gold standard of spinal fusion, a technique first proposed by Briggs H and Milligan P (8), the earliest use of excised laminae as intervertebral space plant fusion, with the development of technology and the rise of various materials, The use of different materials and different types of fusion apparatus and internal fixation in clinical treatment, such as surgical titanium and polyether ether ketone (PEEK), has reduced the failure of internal fixation. In our study, all patients underwent PLIF surgery, pedicle nail rod system fixation and interbody implant fusion device, which was made of polyether ether ketone (PEEK). Titanium cages can improve the rate of bone fusion, but also increase the settlement caused by the hardness of titanium relative to bone. PEEK materials have more skeletal elasticity and exhibit less settling than titanium cages (9). Cage made of different PEEK materials can be selected according to the patient's condition to stretch the vertebral space recovery height (10). Indirect decompression is achieved by stretching the ligaments of the surrounding tissue through the intervertebral height recovered by the fusion apparatus. At the same time, the interbody fusion device was used as a bone graft carrier to achieve interbody fusion and restore stability to the unstable segment (11). From this point of view, the cage apparatus plays a crucial role in interbody fusion surgery.

Cage subsidence is the most common complication after PLIF. However, the development process of cage subsidence is not very clear. Generally speaking, the most direct impact of cage subsidence is that it can cause the intervertebral height to drop, thus weakening the supporting force of the anterior column of the spine, indirectly causing changes in the soft tissue structure reconstructed during surgery, such as ligament relaxation and hypertrophy and compression of the spinal cord or nerve root, thus affecting the decompression effect of surgery. It can affect the improvement of postoperative clinical symptoms and the long-term prognosis of surgery, but there are still many opinions and opinions about the relationship between postoperative clinical symptoms and fusion device subsidence. When exploring the correlation between fusion sink and clinical outcome during posterior lumbar interbody fusion, Oh et al. (12) retrospectively analyzed 102 patients and followed them up for 1 year. They found that fusion sink was correlated with bone mineral density, but there was no significant correlation between fusion sink and deterioration of clinical symptoms. In a study conducted by Choi et al. (13) on the stage of postoperative subsidence, the relationship between fusion, symptom recurrence and subsidence development was evaluated on imaging data, and the conclusion was that subsidence had no direct relationship with symptom recurrence and imaging fusion. The results of these studies are the same as the results of this study. During the follow-up period, fusion device sedimentation did not significantly affect the VAS score and ODI score, which may be directly related to drug treatment, small sample size and short follow-up period of patients. However, there are also many scholars who believe that the interbody fusion device subsidence will significantly affect the postoperative results of patients. Marchi et al. (14) pointed out in the study report that after fusion, fusion device subsidence would lead to transient low back pain in the early postoperative period, and this symptom would be relieved as the fusion device gradually stabilized. Lewandrowski et al. (15) reported a patient with interbody fusion organ subsidence. They believed that the subsidence reduced DH, resulting in soft ligament relaxation and further compression of nerve roots, leading to symptom recurrence. In a meta-analysis, Macki reported that the rate of surgical revision due to interbody fusion vessel subsidence was 2.8%. Athan et al. (16), when studying the risk factors of subsidence after anterior lumbar interbody fusion, pointed out that fusion sink was significantly correlated with adjacent segment degeneration and the incidence of secondary surgery. Postoperative fusion subsidence can affect the effect of indirect decompression and spinal stability, and may cause local inflammation resulting in postoperative low back pain (14). It can be seen that regardless of the influence of fusion device subsidence, preventing the occurrence of postoperative subsidence is still of great clinical significance for maintaining sagittal balance and optimizing surgical prognosis. By analyzing various factors, this study further analyzed the high-risk factors causing fusion device subsidence and clarified their correlation.

At present, the clinical research on the cage subsidence mostly starts from the stress balance and biomechanics between the fusion device and the end plate. At present, it is believed that the settling of the fusion plate is mainly caused by the uneven pressure of the fusion plate between the vertebrae. Once the bearing capacity of the vertebrae is exceeded, the end plate will be damaged and the fusion plate will migrate. The uneven load is an important factor leading to the settling of the fusion plate. The settlement does not progress. In terms of its own factors, bone mineral density is generally considered to be an important factor affecting the settlement of the fuse, and the relationship between bone mineral density and end plate strength has become more and more clear. Hou et al. (17) found through biomechanical tests that bone mineral density was closely related to the destructive load of the end plate of the vertebral body, and the decrease in bone mineral density could lead to the decrease in the destructive load of the end plate, thus increasing the risk of interbody fusion. Liu Lei et al. (18) also found that bone density was closely related to the height of fusion sink, and the lower the bone density, the greater the possibility and height of fusion sink. In this study, it was also found that bone density was closely related to fusion sink, and the better the bone density, the lower the possibility of fusion sink. Rafael et al. (19) found in their experiments that anti-osteoporosis treatment could significantly promote bone fusion and reduce the risk of fusion sink. Therefore, it is recommended that surgeons conduct bone mineral density detection before interbody fusion, and apply anti-osteoporosis treatment to reduce the incidence of postoperative interbody fusion subsidence when osteoporosis is found. In addition, BMI, gender and age are also considered to be relevant self-influencing factors. Chen et al. (20) pointed out in their study that women and body mass index are also risk factors for fusion device subsidence. Yao et al. (21), in a study of fusion after minimally invasive transforaminal lumbar interbody fusion, found that the BMI of patients in the sedimentation group was higher than that in the control group. Marchi et al. (14) pointed out in their report that they believed there was a certain correlation between the age of patients and the severity of subsidence, but did not clearly point out whether age was associated with the occurrence of subsidence. However, in this study, it was found that gender, BMI, and age were not significantly correlated with the fusion chamber settlement. Similarly, Beutler et al. (22) said in a study of 104 patients that the patient's BMI, age, gender and other factors could not significantly affect the fusion chamber settlement, and there was no statistical significance. At the same time, the smoking history, alcohol history and basic diseases of the patients were also studied in this study, and there was no significant significance to the fusion chamber subsidence. Although the relationship between self-factors such as BMI, gender, age and underlying diseases and fusion subsidence is not clear, overweight or obesity may affect the prognosis of surgery, the influence of age on bone mineral density should not be ignored, and smoking affects interbody fusion time, which may indirectly affect fusion subsidence. Therefore, in the perioperative evaluation, we should pay attention to the patient's age, weight and other factors, and make good preparation for preoperative education and operation.

In addition to the patient's own factors, surgical skills and methods are also the key factors of fusion device subsidence. Intervertebral height can also affect the outcome of fusion surgery. Arun-Kumar et al. (23) found in a study on the factors affecting the early disc height decline after lateral lumbar interbody fusion that the greater the increase in interbody height during surgery than before surgery, the greater the loss of interbody height during the entire early follow-up. Therefore, they believed that the optimal disc height, for example, by referring to the height of the interbody space before surgery, should be obtained. It is an important surgical strategy rather than an excessive pursuit of increased vertebral space height. Yang et al. (24) pointed out in their study that excessive spacing of the vertebral space would increase the stress in the fusion stage and accelerate the onset of settlement. Intraoperative vertebral space correction height should be selected according to the patient's preoperative degeneration and vertebral space height. In this study, it was found that when the vertebral space correction height was too large, the probability of fusion would be increased, especially in patients with more severe degenerative diseases and more rigid anatomical structure. Based on these findings, surgeons should emphasize optimizing the size and intervertebral height adjustment of the fusion vessel to reduce the risk of end plate injury and fusion vessel subsidence. In addition, it is generally believed that how to deal with the end plate in fusion surgery is a powerful factor that cannot be ignored. The ultimate goal of interbody fusion is to release the nerve or spinal cord compression from intraspinal lesions and restore the intervertebral high-maintenance spinal sequence. The integrity of the vertebral end plate is an important condition to ensure the support strength of the end lamin-fusion contact surface, and excessive damage to the bony end plate will increase the risk of subsidence. Intraoperative end plate damage reduces the end plate failure load, resulting in fusion sink (25). WEWEL et al. (26) pointed out in their study the importance of intraoperative end plate protection to prevent fusion device subsidence, and this study also found that end plate damage is a risk factor for fusion device subsidence. Current mainstream studies support reducing end plate damage during surgical operations as much as possible in order to reduce the risk of fusion device subsidence. The relationship between the Modic parting and the fusion settlement is not clear, but considering the different end plate failure load under different parting may have an effect on the fusion settlement. Among them, type III is the hardening of end plate cartilage, which hardens the vertebral body and increases the destructive load of the end plate, which may reduce the probability of fusion. Clinical studies have also pointed out that segmental lordosis can be an independent risk factor for fusion sink (18). Excessive correction of segmental lordosis can lead to separation of the front half of the fusion and the end plate. Biomechanical studies have shown that the small contact area between the fusion and the end plate is one of the reasons for fusion sink (27). The excessive correction segmental convex Angle causes the stress to be concentrated in the rear part of the fusion plate, resulting in a high risk of fusion settlement. However, the Angle of segmental lordosis correction is not clear in clinical studies. In this study, it was found that attention should be paid to the reconstruction of local lordotic Angle and lumbar lordotic Angle during surgery to avoid excessive extension of the intervertebral space and construction of excessive SL Angle, so as to avoid the stress affecting the fusion segment and the acceleration of sedimentation (28). Proper handling of the intervertebral space during surgery and finding the appropriate degree of SL improvement can reduce the rate of reoperation. In addition, studies have found that PI-LL is also a risk factor for fusion device subsidence. Schwab et al. (29) proposed that PI-LL < 10° is spinal pelvic matching, which has important significance for spinal stability. Clinically, PI-LL was divided into three degrees: < 10°, 10°–20° and > 20°. The higher the PI-LL value, the greater the likelihood of postoperative complications of lumbar spine. The incidence of the latter two degrees was 1.1 times and 5.3 times of PI-LL < 10°, respectively (30). In addition, Masevnin et al. (31) reported that PI-LL mismatch is a risk factor for spinal sagittal instability, and patients with high PI value and low LL value have a significantly increased risk of fusion instrument subsidence after short-segment fusion. After operation, unreasonable sagittal position parameters will affect the stability of the spine and the mechanical structure will change, which will affect the fusion device settlement, but there is still a lack of clinical research in this aspect. The surgeon should reasonably restore the height of the intervertebral space and correct the segmental lordosis Angle according to the patient's degeneration and sagittal position parameters, so as to reduce the probability of fusion device subsidence.

Clinical studies on the risk factors of fusion are detailed, but the correlation between paravertebral muscle and intervertebral bone fusion time and fusion has not been reported. In this study, the CSA and FI of the paravertebral and psoas major muscles were measured, which are key parameters for evaluating paravertebral atrophy (32). The paravertebral muscle is an important muscular system that helps stabilize the spine during the maintenance of normal lumbar physiological lordosis and dorsal extension (33). In severe paraspinal atrophy, spinal stability is reduced and stress between the fusion plate and the end plate is constantly changing, resulting in fatigue damage to the end plate resulting in fusion plate subsidence (34). Singhatanadgige et al. (2) found paravertebral muscle atrophy to be a risk factor for fusion sink in the follow-up of patients undergoing MIS-TLIF surgery. In this study, it was found that paravertebral muscle rFCSA was an independent risk factor for fusion. The pulling effect of paravertebral muscle on the posterior structure of the vertebral body could reduce the stress on the fusion, and the stress between the fusion and the end plate increased when the paravertebral muscle atrophy. Wang Sinian et al. (35) also pointed out that paravertebral muscle degeneration is closely related to sagittal position parameters, and the paravertebral muscle affects the sagittal position force line to maintain spinal sagittal balance through a compensatory mechanism. The larger the paravertebral muscle rFCSA was, the less likely the fusion sink was to occur. However, the biomechanical relationship between the paravertebral muscle and the fusion apparatus needs further study. The functional exercise of paravertebral muscle is related to the prognosis of surgery. Having good muscle strength can obtain better curative effect and fewer complications after surgery. In addition to drug assisted treatment, clinicians should also give reasonable muscle function exercise methods. In addition, the time of interbody fusion is also closely related to fusion sink. Lee et al. (36) found in the follow-up of 79 patients undergoing spinal fusion surgery that when interbody fusion occurred and the new bone could withstand the load at the interbody fusion-vertebral end plate interface, the settlement would no longer progress. Prolonged intervertebral fusion can lead to osteolysis and absorption in the implanted bone of the intervertebral space (37). After osteolysis and absorption, the stress between the fusion organ and the end plate of the vertebral body increases, and the fusion organ subsidence occurs when the load exceeds the end plate failure. When the fusion apparatus sinks, the instability of the intervertebral space will further affect the intervertebral fusion time. The two can influence each other and cause and effect each other. When the intervertebral fusion time is prolonged, the intervertebral bone fusion is poor, and good bone support cannot be formed, and the load will be too much relied on the installation of the pedicle internal fixation to bear the load. Long-term high load will lead to the fatigue of the metal internal fixation, resulting in the fracture of the screw or rod (38). Failure of internal fixation will also increase the stress between the fusion-end plate of the vertebral body, resulting in the settlement or even displacement of the fusion, which will affect the surgical effect and require a second surgical repair in severe cases. Therefore, osteogenic drugs should be actively used after surgery to reduce the time of intervertebral bone fusion and reduce the possibility of fusion sink.

The surgeon should reasonably avoid or reduce the influence of high risk factors before surgery, reduce the probability of postoperative fusion device subsidence, and achieve better surgical efficacy and prognosis.

## Limitations

The patients included were all undergoing single-stage fusion surgery, and the effect of the number of fusion segments on the fusion sink was not studied. Because the width and other specifications of the fusion device used by the patients are the same, the influence of the fusion device specifications on the settlement is not clear. This study is a single-center study with a small sample size, and the results are accidental and less persuasive.

## Conclusions

**Fig. 2:**
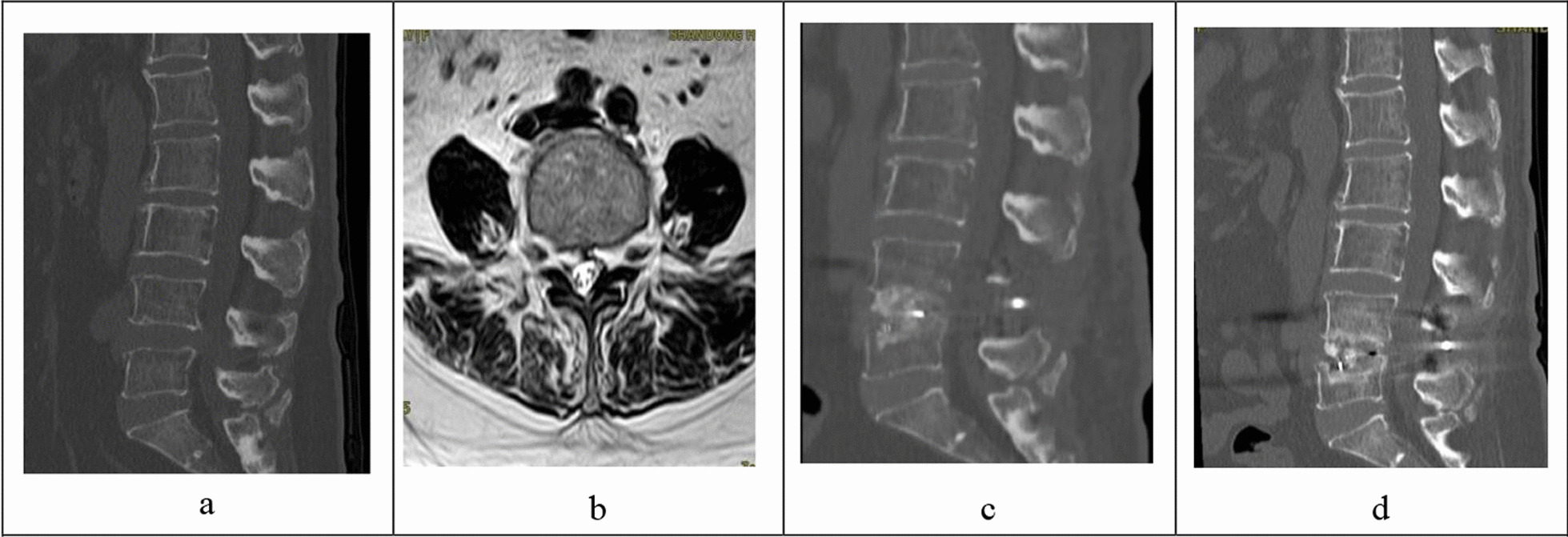
A 64-year-old female patient was admitted to hospital with "lumbar disc herniation". Her bone mineral density (BMD) was − 1.7 g/cm^2^. PLIF surgery was performed, and the fusion device settlement was 4.46 mm after surgery. **a**: Preoperative CT of lumbar spine showed that the SL of L4/5 was 2.63°, and the upper and lower endplates were intact. **b**: Preoperative horizontal MRI under L4 end plate showed that the rCSA of the psoas major was 1.32, the rCSA of the paravertebral muscle was 3.18, the rFCSA of the paravertebral muscle was 1.77, and the vertebral area was 1367.28mm2. **c**: Sagittal CT scan of lumbar spine 1 day after surgery showed intraoperative end plate destruction, the correction height of intervertebral space was 1.71 mm, the correction Angle of SL was 7.13°, and the PI-LL was 23.76°. **d**: Sagittal CT scan of the lumbar spine 8 months after surgery showed poor intervertebral fusion, osteolysis and absorption, and significant fusion sink

**Fig. 3:**
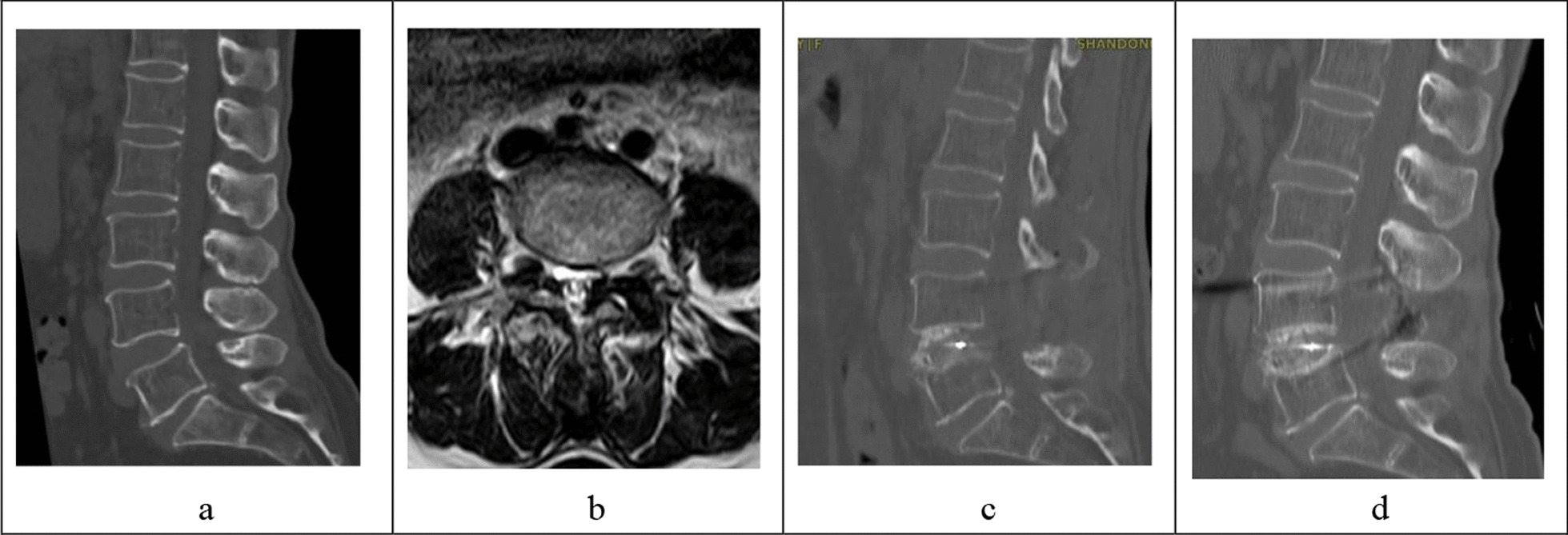
A 74-year-old female patient was admitted to hospital with "lumbar spinal stenosis". Her bone mineral density (BMD) was − 2.6 g/cm^2^. She underwent PLIF surgery and the fusion sink was 3.14 mm after surgery. **a**: Preoperative CT of the lumbar spine showed that the SL of L4/5 was 12.85°, and the upper and lower endplates were intact. **b**: Preoperative end plate horizontal MRI under L4 showed that the rCSA of the psoas major muscle was 1.29, the rCSA of the paravertebral muscle was 3.31, the rFCSA of the paravertebral muscle was 2.28, and the vertebral area was 1254.19 mm^2^. **c**: 1 day after surgery, sagittal CT of lumbar spine showed that the height of intervertebral space correction was 4.01 mm, the Angle of SL correction was 3.61°, and the PI-LL was 3.74°. **d**: 1 year after surgery, sagittal CT of the lumbar spine showed good intervertebral fusion and significant fusion sink

In summary, the risk factors for lumbar fusion are complex and diverse. Among them, the height of immediate intervertebral space correction, the Angle of immediate SL correction and the time of intervertebral bone fusion can all be independent risk factors for fusion, while good bone density and paravertebral muscle rFCSA can play a preventive role in fusion. Surgeons should consider the high risk factors before operation and in operation design, and actively treat the symptoms. Intraoperative technical practice to reduce intraoperative end plate injury (Figs. 2, 3).

## Data Availability

The datasets generated and analyzed during the current study are available from the corresponding author on reasonable request.
